# Does Electric Friction Matter in Living Cells?

**DOI:** 10.1021/acs.jpcb.1c02783

**Published:** 2021-06-03

**Authors:** Dmitrii E. Makarov, Hagen Hofmann

**Affiliations:** † Department of Chemistry and Oden Institute for Computational Engineering and Sciences, University of Texas at Austin, Austin, Texas 78712, United States; ‡ Department of Chemical and Structural Biology, Weizmann Institute of Science, 76100 Rehovot, Israel

## Abstract

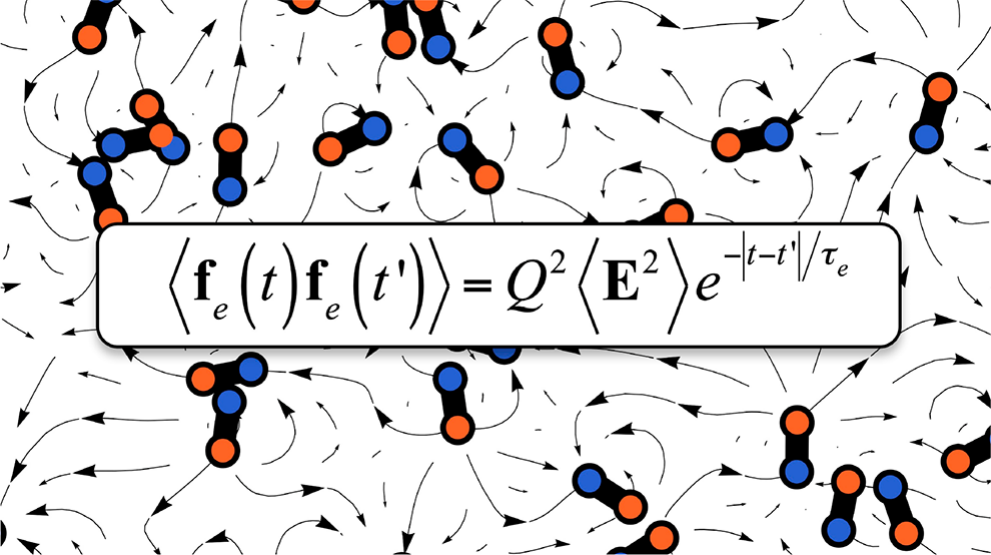

The thermal motion of charged proteins causes randomly fluctuating electric fields inside cells. According to the fluctuation–dissipation theorem, there is an additional friction force associated with such fluctuations. However, the impact of these fluctuations on the diffusion and dynamics of proteins in the cytoplasm is unclear. Here, we provide an order-of-magnitude estimate of this effect by treating electric field fluctuations within a generalized Langevin equation model with a time-dependent friction memory kernel. We find that electric friction is generally negligible compared to solvent friction. However, a significant slowdown of protein diffusion and dynamics is expected for biomolecules with high net charges such as intrinsically disordered proteins and RNA. The results show that direct contacts between biomolecules in a cell are not necessarily required to alter their dynamics.

## I Introduction

The cell is densely filled with proteins, RNAs, and metabolites.^1^ Many studies in the past have investigated how the cellular interior affects the stability and dynamics of proteins.^2^ Clearly, the excluded volume of numerous intracellular macromolecules (i.e., crowding) can substantially impact the behavior of proteins. In fact, protein concentrations can reach 300 g/L, thus proteins may occupy 30% of the cell volume.^3^ As a result, translational diffusion slows down and becomes anomalous,^4^ intrinsically disordered proteins (IDPs) become more compact,^5^ and folded proteins are expected to be stabilized.^6^ Whereas excluded volume crowding has long been the focus of theoretical approaches,^7^ other interactions between biomolecules, sometimes called quinary interactions, have received less attention even though they may not be negligible.^3a,8^

Electrostatic interactions are particularly relevant owing to their long-range nature. Given that most biological macromolecules carry charges, we would expect nonspecific electrostatic interactions to play an important role inside cells. In fact, intramolecular charge–charge interactions sensitively alter the conformation of intrinsically disordered proteins (IDPs).^9,10^ Electrostatic forces, however, also act on much greater length scales, with consequences for the dynamics of biomolecules. For example, previous simulations found a slowdown in the diffusion of a charged protein in the presence of charged cytosolic biomolecules.^11^ Although charge–charge interactions can cause a nonspecific “sticking” of proteins to other cytosolic compounds, here we are concerned with a more general question: do electric field fluctuations caused by cytosolic macromolecules affect the diffusion of a charged particle?

**Figure 1:**
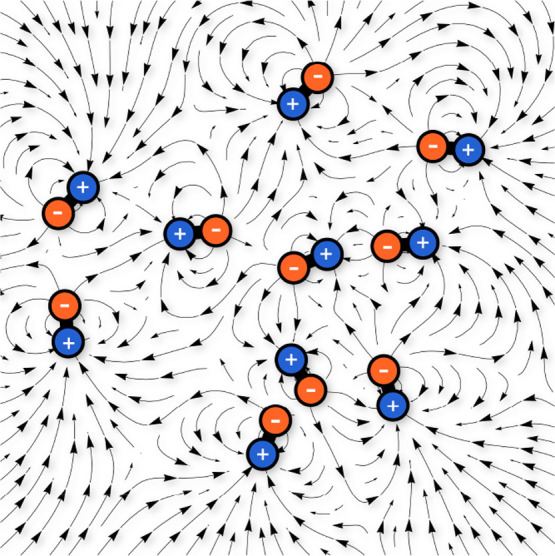
Proteins in the cytosol are diffusing and tumbling dipoles that create a randomly fluctuating electric field. The scheme illustrates the force directions experienced by a charged tracer in a random 2D arrangement of dipoles.

To answer this question, we study the impact of fluctuating electric fields (Figure 1) on the translational diffusion of a charged Brownian particle and on the dynamics of a charged Rouse chain. Following previous approaches,^12^ we assume that electric field fluctuations are well-approximated by colored Gaussian noise with a finite correlation time, which is associated with an electric friction memory kernel. Whereas proteins are by far the dominating macromolecules in cells,^1^ they carry only moderate net charges on average.^13^ Electric friction effects are therefore unlikely to arise from protein net charges. Protein dipole moments, on the other hand, are substantial.^14^ The thermally driven tumbling and diffusion of the enormous number of cytosolic proteins therefore appear to be a more likely source of electric friction (Figure 1). Notably, the cytosol also contains significant amounts of RNA,^1^ which is highly charged and also has the potential to cause electric friction. However, electric friction due to polyions has been studied previously^12,15^ and is not considered in our study.

In the following, we derive an explicit expression for the electric friction memory kernel that is caused by randomly orienting protein dipoles. We demonstrate that the slowdown of translational diffusion of a charged tracer protein due to electric friction is generally modest. However, protein tracers with high net charges may be significantly affected by electric friction. In addition, we provide an estimate of how electric friction impacts the conformational dynamics of charged polymers and find that it has similarities to the previously studied internal friction^16^ in IDPs.

This article is partitioned into two sections. In section II.A, we introduce a description of electric friction based on a generalized Langevin equation. We derive an expression for the magnitude of the electrostatic force fluctuations and, using the model in which these fluctuations are described as colored noise with a finite correlation time, further derive an expression for the friction memory kernel. We then use these results to estimate how electrostatic fluctuations affect the diffusion coefficients of charged particles. In section II.B, we include electric friction in a Rouse chain and study how it affects the chain reconfiguration time.

## II Results

### II.A Diffusion of a Charged Particle with Electric Friction

In the absence of electric friction (to be considered below), the diffusion of a tracer particle in a liquid can be described by the Langevin equation (LE). Assuming an isotropic and homogeneous medium, it suffices to consider the LE in one dimension
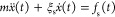
1Here, *x*(*t*) describes the time evolution of the *x* component of the particle’s position, *ẋ*(*t*) = *v*(*t*) = d*x*(*t*)/d*t* is the velocity of the particle, *m* is its mass, *ξ*_s_ is the solvent friction coefficient, and *f*_s_(*t*) is a Gaussian-distributed, delta-correlated random force that satisfies the fluctuation–dissipation theorem
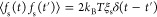
2where *k*_B_ is Boltzmann’s constant, *T* is the temperature, and δ is the Dirac delta function. A well-known consequence of eqs 1 and 2 is the Stokes–Einstein relationship that relates the macroscopic drag force characterized by *ξ*_s_ to the microscopic diffusion coefficient *D* via
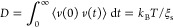
3Randomly tumbling and diffusing proteins in the cytosol will give rise to an additional fluctuating electrostatic force *f*_e_(*t*), which would have to be added to the rhs of eq 1. According to the fluctuation–dissipation theorem,^17^ there is an associated friction force such that the resulting equation of motion is of the form of the generalized Langevin equation^12a^ (GLE)
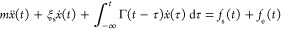
4Here, the electric friction memory kernel Γ(*t*) satisfies the fluctuation–dissipation theorem
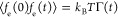
5Importantly, in contrast to solvent friction, which is caused by collisions of solvent molecules with the tracer particle at picosecond time scales, the time scales associated with the tumbling and diffusion of macromolecules are relatively long. As a result, the random force *f*_e_ cannot, in general, be viewed as delta-correlated, so the GLE is an integro-differential equation.

If the fluctuations of the electric field **E** can be described by a single characteristic time scale *τ*_e_, then its autocorrelation function can be approximated by an exponential function,
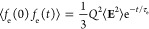
6Here, *Q*^2^⟨**E**^2^⟩ is the mean-squared electrostatic force that acts on a diffusing particle with charge *Q*. (Note that ⟨**E**⟩ = 0 for a randomly tumbling dipole.) As a result, the friction memory kernel is also an exponential function,
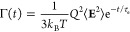
7Given the heterogeneous cytosol composition with macromolecules of different sizes and shapes together with the time scale separation between tumbling and translational motions, this single-exponential approximation is unlikely to be accurate but can easily be generalized to include multiple exponentially decaying terms with different characteristic time scales.^18^

Within the exponential memory kernel approximation, the electric friction is characterized by two parameters: the strength of the electric field fluctuations ⟨**E**^2^⟩ and the memory time *τ*_e_. When the time scale of interest is much longer than *τ*_e_, however, the effect of electric friction can be characterized by a single parameter, the electric friction coefficient
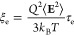
8This parameter corresponds to the Markovian approximation to the GLE, where the friction memory kernel is approximated by the delta function, Γ(*t*) ≈ 2δ(*t*) *∫*_0_^∞^ Γ(τ) dτ = 2*ξ*_e_*δ*(*t*), and thus the electric friction force in eq 4 is approximated simply by −*ξ*_e_*ẋ*(*t*). Hence, the friction coefficient defined in eq 8 is usually only meaningful for sufficiently short *τ*_e_. In general, it can be shown that an exponential memory kernel (eq 7) results in a modified Stokes–Einstein relationship, which depends on *ξ*_e_ alone and not on *τ*_e_:
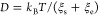
9See the Appendix for a derivation of eq 9 from eqs 3, 4, and 7.

To estimate the electric friction memory kernel, we need the magnitude of the electric field fluctuations ⟨**E**^2^⟩ resulting from the tumbling and diffusion of cytosolic protein dipoles. To compute ⟨**E**^2^⟩, we consider the electric field produced by a dipole formed by charges ±*q* located at **r**_±_ and separated by a distance *l* = |**r**_–_ – **r**_+_|. The electrostatic potential experienced by a charged tracer particle located at the coordinate origin is given by
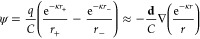
10Here, *r*_+_ = |**r**_+_| and *r*_–_ = |**r**_–_| are the distances between the diffusing particle and the positively and negatively charged ends of the dipole, respectively, **d** = *q* (**r**_–_ – **r**_**+**_) is the dipole moment vector with magnitude |**d**| = *d*, κ is the inverse Debye screening length, and *C* = 4*πϵ*_0_*ϵ*_w_ with the dielectric constant of water *ϵ*_w_ = 80 and the vacuum permittivity ϵ_0_. Assuming that the dipole length *l* is much smaller than the distance *r* between the dipole and the particle, one finds
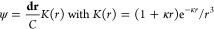
11The electric field is **E** = −∇ψ, and its mean-square magnitude ⟨**E**^2^⟩ is obtained by averaging over all angles θ and positions *r*, thus leading to
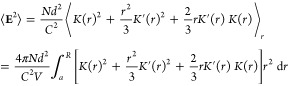
12where the prime indicates the derivative with respect to *r* (Appendix IV.B). Here, *R* is the radius of a spherical cell, *V* ∝ *R*^3^ is the cell volume, and *N* is the number of dipoles in a cell. The lower integration bound (*a*) for the positional distribution must be chosen such that the tracer particle does not collide with the dipole (*a* > *l*/2); in fact, the dipole approximation used to arrive at eq 11 would even require *a* ≫ *l*/2. To understand how the magnitude of force fluctuations depends on the system size and the dipole concentration, we first consider the case without screening (κ → 0) and then discuss the effects at physiological ionic strengths. Under the assumption that the cell is much larger than the dipole, we find
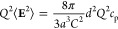
13for the force fluctuations and
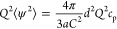
14for the energy fluctuations. Here, *c*_p_ = *N*/*V* is the concentration of dipoles (proteins) in a cell. Notably, the fluctuation magnitude is independent of the cell size *R* (which provides a justification of the above spherical symmetry assumption as long as *R* is large enough) and scales linearly with the concentration of dipoles. Hence, despite the long-range nature of electrostatics, only neighboring dipoles effectively contribute to the force amplitude. In addition, the mean-squared fluctuation amplitude and therefore the electric friction coefficient increase with the squared charge of the tracer particles (*Q*^2^), which may lead to significant contributions for highly charged particles. The expressions in the presence of salt-screening (i.e., at arbitrary κ) are given in Appendix IV.B and were used in all calculations.

**Figure 2:**
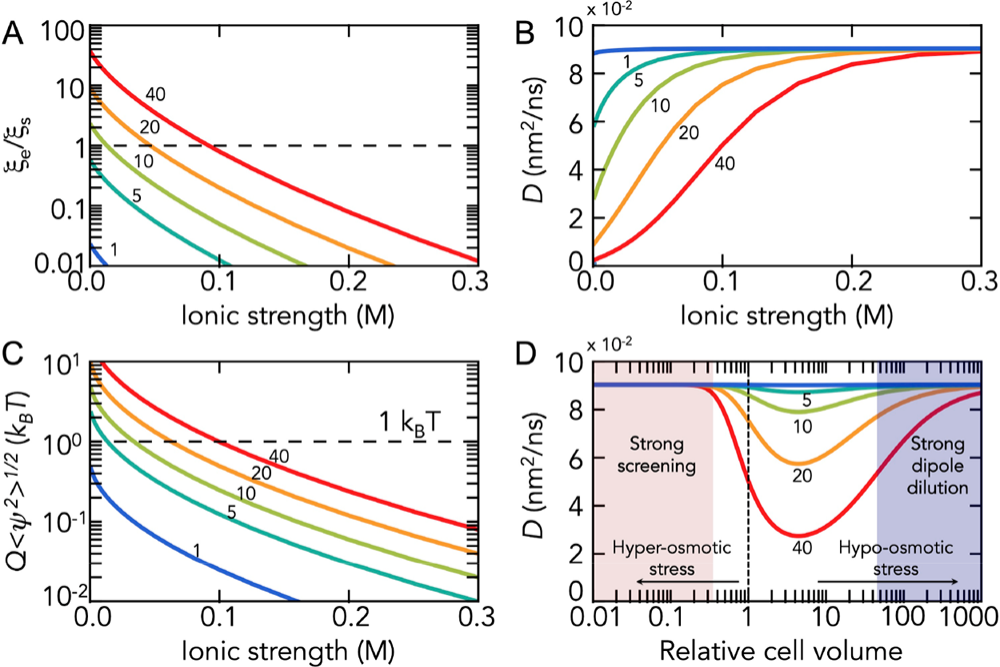
Effect of electric friction on the diffusion of a charged particle (net charge number indicated). (A) The electric friction coefficient (*ξ*_e_) relative to solvent friction (*ξ*_s_), (B) the diffusion coefficient, and (C) the energy magnitude of electrostatic fluctuations are moderately impacted for diffusing particles with a net charge of *Q* < 5e. Significant effects on particle diffusion at physiological ionic strengths (0.1–0.3 M) are observed only for highly charged particles. Calculations were performed with the following parameters: *c*_p_ = 6 mM, *a* = 3/4 *l*, *l* = 4.8 nm, and *d* = 635 D. (D) A change in cell volume simultaneously changes the ionic strength and dipole concentration. At low cell volumes (i.e., high ionic strength and dipole concentration, red area) and high cell volumes (i.e., low ionic strength and low dipole concentrations, blue area), the effect of electric friction is negligible. In the intermediate regime, we would expect a slowdown of the diffusion of a charged tracer particle if the cell volume increases compared to its natural value (*V* = 1).

With eqs 8 and 12, we can now compute the ratio between electric friction (*ξ*_e_) and solvent friction (*ξ*_s_) for realistic values of *c*_p_, *d*, and *a*. A previous study computed the dipole moments of 11 981 proteins from the protein database and obtained a mean dipole moment of 639 D (median = 452).^14^ The average size of a bacterial protein is 267 amino acids,^19^ which results in a Stokes radius of 2.4 nm using the experimental scaling laws for folded proteins.^20^ The dipole length is therefore *l* ≈ 4.8 nm, and we took *a* = 3*l*/4. The protein concentration inside a bacterial cell is ∼6–11 mM (for a randomly tumbling dipole), and we assume a cell volume of 1 fL. Because *ξ*_e_ ∝ *τ*_e_ (eq 8), we also require the time scale *τ*_e_, which will significantly impact the magnitude of electric friction. The electric field fluctuations at any given point are caused by the rotational and translational motions of the dipoles, and we therefore expect the friction memory kernel (eq 5) to display time scales ranging from those of tumbling (nanoseconds) to diffusion (milliseconds). A conservative estimate would use the lowest possible time scale (dipole tumbling), which is given by *τ*_e_ ≈ *vη*/*k*_B_*T* with *v* ≈ (*l*/2)^3^ for the volume of a dipole with η being the viscosity. We assume that the dipoles tumble independently and experience only the drag force of the solvent that is defined by the viscosity η (1 mPa s). Given these numbers, we find that the relative impact of electric friction decreases drastically with increasing ionic strengths (Figure 2A). For moderately charged particles (|*Q*| < 5e), the electric friction coefficient is several orders of magnitude smaller than solvent friction at physiological ionic strengths (100–300 mM) (Figure 2A). This result is in agreement with the electrostatic energy fluctuations that reach only thermal energy at high net charges (Figure 2B). Given that the net charges of most proteins rarely exceed 5e, we generally conclude that electric friction does not affect the translational motion of proteins significantly. However, highly charged proteins such as histone H1 (net charge +53e) and prothymosin-α (net charge −44e)^21^ are likely affected, even at physiological salt concentrations, and their diffusion coefficients can decrease 2-fold (Figure 2C). Importantly, although electric friction seems to be a rather moderate effect compared to other contributions in the cytosol such as direct protein–protein interactions, it should be kept in mind that our results are approximate since the contribution of highly charged RNA as well as fluctuations due to charge regulation^22^ such as protonation and deprotonation of ionizable groups were neglected. With these conservative estimates, electric friction is just on the verge of affecting the diffusion of charged proteins in a cell and a more accurate treatment using simulations might discover more pronounced effects. We note that our results can also provide estimates for other scenarios, such as liquid protein condensates with protein concentrations (500 g/L)^23^ that even exceed those found in cells.

How can electric friction be identified experimentally? Clearly, disentangling electric friction from other effects such as excluded volume crowding or “sticking” is challenging, yet compared to processes that require direct physical contacts, electric friction has an unusual dependence on the cell volume within the limit given by our assumption of a fixed time scale of electric field fluctuations. Although electric friction does not explicitly depend on the volume of a cell, altering the cellular volume by applying osmotic stress will alter the concentration of protein dipoles (eq 13) together with the ionic strength of the cellular interior. Clearly, in the extreme case of strong dilution (Figure 2D) (i.e., at large cell volumes), electric friction is negligible. Remarkably, the same phenomenon is observed at extremely small cell volumes (Figure 2D). Although both dipole concentration and ionic strength are high at small cell volumes, the high ionic strength effectively screens charge interactions such that electric friction remains marginal. In the intermediate regime, electric friction has a maximum. Particularly for the reported cellular concentrations of proteins (6 mM) and ionic strength (0.15 M), a 2-fold increase in cell volume would cause an increase in electric friction (i.e., a decrease of the diffusion coefficient of a charged tracer particle (Figure 2D)). On the contrary, excluded volume effects and/or direct interactions of the particle with cytosolic components would rather cause a slowdown in diffusion. Recent measurements by König et al. indeed found a slowdown in the translational diffusion of a charged disordered protein under hyperosmotic conditions,^24^ which would suggest that crowding and “sticking” dominate inside cells. We note, however, that our estimate for the volume dependence of electric friction is valid only under the assumption that the time scale of electric field fluctuations (*τ*_e_) is invariant under volume changes. Clearly, a dipole in a cell experiences the field of neighboring protein dipoles such that volume changes will also affect the average tumbling time.

### II.B Rouse Chain with Electric Friction

The internal dynamics of proteins (i.e., their conformational changes) cover many orders of magnitude in time, from the reconfiguration time scales in unfolded proteins to those of protein folding and large molecular assemblies.^25^ Because proteins function in aqueous solutions, frictional forces determine not only their translational diffusion but also their internal dynamics such as folding. Theories based on Kramers’ model of diffusive barrier crossing^26^ in the overdamped limit predict a direct proportionality between the folding time τ and viscosity (τ ∝ η),^27^ which has indeed been observed for the millisecond folding dynamics of two-state proteins.^28^ In contrast, deviations from this behavior were found for proteins that fold within microseconds^29^ or that exhibit significant ruggedness in their energy landscapes.^27b^ Two alternative ways of analyzing these experimental data have been used in the past. Either a friction component resulting from interactions within the protein has been invoked, which leads to an additive time scale that is independent of the viscosity of the solvent,^27b,29a^ or if a scaling relationship between the reaction time and solvent viscosity is assumed, then a weaker viscosity dependence such as τ ∝ *η*^β^ with β < 1 was used to describe the experimental data.^30^ Indeed, such fractional viscosity dependencies have been identified for the diffusion of small molecular compounds^31^ and the internal dynamics of molecules.^32^ Physical interpretations of such behaviors range from a viscosity-dependent change in the hydrodynamic coupling between the solvent and the molecule^33^ and a breakdown of continuum hydrodynamics due to the finite size of the solvent molecules^31a,34^ to memory effects caused by solvent relaxation or degrees of freedom different from the probed reaction coordinate.^32,35^ Non-Markovian electric friction may also contribute to the complexity of the observed internal dynamics of charged biopolymers.

Thus motivated, we seek to understand the effect of electric friction on the internal dynamics of charged biopolymers. To this end, we consider the dynamics of the simplest polymer model, a Rouse chain. Despite their simplicity, Rouse/Zimm-type models often offer a quantitative description of reconfiguration dynamics of intrinsically disordered proteins^16,36^ and other biopolymers.^37^ Their success is in part due to the fact that they are coarse-grained models capturing specific intermonomer interactions via a few adjustable parameters. Here, for simplicity, we focus on the Rouse model, which ignores hydrodynamic interactions. The usual coarse-graining monomeric unit of the Rouse chain is the Kuhn segment chosen to be sufficiently large such that adjacent Kuhn segments are statistically independent. In the present case, however, it is essential to consider another length scale *l*_C_, which is the length over which the spatial correlations of the electric field acting on the polymer decay. If *l*_C_ is comparable to or smaller than the size of the Kuhn segment, then the electric forces acting on each Kuhn segment are statistically independent. Is this a reasonable assumption?

When taking the nearest-neighbor distance^38^ between two dipoles as an upper limit for *l*_C_, we find 3–3.4 nm at cellular concentrations (6–11 mM) (i.e., much larger than the Cα distance between adjacent amino acids (0.38 nm) and also larger than the Kuhn length (0.55 nm) of polypeptide chains).^39^ Hence, neighboring amino acids will experience the same electrostatic force.

When *l*_C_ exceeds the size of the Kuhn segment but is still much smaller than the radius of gyration of the entire chain, *R*_g_, then it is expedient to further coarse grain the chain to de Gennes “blobs” of size *l*_C_. Because of the self-similarity of the Rouse model, such coarse graining does not affect the model’s dynamic properties (except for short time scales), yet now the electric forces acting on each blob may be considered statistically independent. This case is discussed in detail below. If, however, *l*_C_ is comparable to or greater than *R*_g_, then electric forces on all monomers of the chain are correlated. It is then instructive to consider the limit in which all monomers feel the same electric field. Interestingly, unless the chain is charged uniformly, interaction with the electric field also leads to friction. This case is discussed at the end of this section and in Appendix D.

Consider the case *l*_C_ ≪ *R*_g_ and assume that the problem is adequately course-grained such that the electric forces on each monomeric unit are statistically independent of one another. The chain is then treated as a Rouse chain (i.e., as a string of *n* harmonically coupled beads (blobs)).^40^ A Rouse model with explicit noncovalent contacts between beads has been developed by Barsegov, Morrison, and Thirumalai^41^ but since we mainly focus on interactions of the beads with external electric fields, we implicitly account for electrostatic interactions between chain beads by the spring constant of the interbead interaction (which may depend on the ionic strength). In addition to the solvent friction (and the associated delta-correlated random force), each bead also experiences an electric friction force along with the stochastic force caused by the fluctuations in the electric field. These forces depend on the charges of each bead, and in general, a numerical solution of the problem can be obtained for any given value of such charges. To ensure that the problem is tractable analytically, however, here we will consider the case in which all of the monomer charges are the same.

The equation of motion for the *x* coordinate of the *k*th bead is thus given by the GLE (cf. eq 4) with two modifications. First, it now includes the interaction force between the beads, and second, we (conventionally) neglected the inertia of the beads that is important only at very short time scales.^42^ The resulting overdamped GLE is
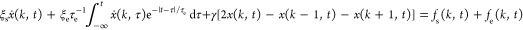
15

Here, the spring constant (γ) is given by
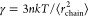
16where ⟨*r*_chain_^2^⟩ is the mean square end-to-end distance of the chain.^16a^ Importantly, eq 15 is not specific to electric friction. Any fluctuating noise source with an exponentially decaying memory kernel will lead to the same formulation, and the following conclusions are general.

We are interested in computing the average reconfiguration time of the chain. Following previous approaches,^16,42^ we define this reconfiguration time by the correlation function *C*_*k*_(*t*) of the vector that connects a bead at position 0 with another bead at position *k*
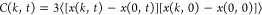
17The mean reconfiguration time of the chain is given by
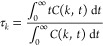
18After switching from bead numbers (*k*) to modes (*q*) using Fourier transform (i.e., neglecting effects from the chain termini), eq 15 becomes
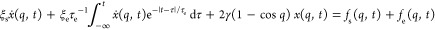
19Here, *q* = 2π{0, 1, 2, . . .}/*n* for a periodic chain. The solution is obtained after reducing eq 19 to a second-order differential equation and using time Fourier transform (Appendix IV.C)
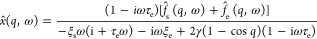
20In contrast to a Rouse chain, the introduction of colored noise results in two relaxation times (τ_1_ and τ_2_) that are given by the two eigenvalues (ω_1_ and ω_2_) of eq 20
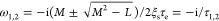
21with *M* = *ξ*_s_ + *ξ*_e_ + 2*γτ*_e_(1 – cos *q*) and *L* = 8*ξ*_s_*τ*_e_*γ*(1 – cos *q*). Using eq 20 together with the fluctuation dissipation theorems (Appendix IV.C), the correlation function is
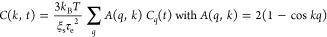
22and the mode number is *z*. The mode-specific correlation function in eq 22 is a double-exponential function given by
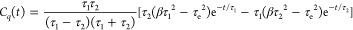
23Here, *τ*_e_ is the tumbling time of the dipoles as defined in section A and β = 1 + *ξ*_e_/*ξ*_s_. Using eqs 18 and 23, we arrive at the average mode-dependent relaxation time of the chain
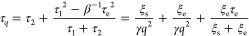
24where we used (1 – cos *q*) ≈ *q*^2^/2. In the limit of the vanishing electric friction (i.e., *ξ*_e_ → 0), we find *τ*_*q*_ = *ξ*_s_/*γq*^2^, which is the relaxation time scale spectrum of the classical Rouse model without electric friction. Notably, the additional friction component adds two contributions to the standard Rouse mode relaxation times. The first component is mode-dependent (second term in eq 24), and the other component contributes equally to all modes (third term in eq 24). These two contributions are also of interest for understanding internal friction processes in polymers. For example, conflicting observations have been reported for the mode dependence of internal friction.^16a^ Whereas single-molecule FRET experiments were in agreement with a mode-independent contribution of internal friction,^16b^ experiments on long polymers suggest a mode dependence of internal friction.^43^Equation 24 combines both types of contributions. In particular, term *q*^–2^ is proportional to the length of the chain squared (*n*^2^) such that the second term in eq 24 will dominate for long chains whereas the mode-independent term (third term) is more important for short chains.

**Figure 3:**
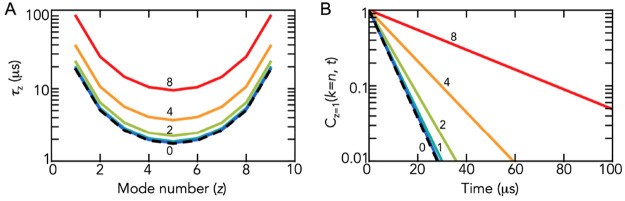
Effect of electric friction on the reconfiguration dynamics of a Rouse chain. (A) Mode spectrum of a chain with *n* = 10 blobs and a blob size of 100 monomers. Mode number *z* is given by *z* = *nq*/2π at increasing net charges of the chain monomers (indicated). The spectrum for an uncharged Rouse chain is shown as a black dashed line. (B) Mode-specific end-to-end vector correlation function (*z* = 1) with *n* = 10 at increasing net charges of the chain beads (indicated). The color code is identical to that in A.

In the specific case of electric friction considered here, the additional friction component causes a slowdown of the chain dynamics across the whole mode spectrum (Figure 3A). In addition, the correlation functions have two decay components, both of which depend on electric friction (eq 21). Yet, the slower component (τ_2_) dominates the correlation function, thus causing a nearly exponential decay (Figure 3B).

Now consider the limit *l*_C_ ≫ *R*_g_. In this case, all monomers of the chain will experience the same external electric field. For a uniformly charged chain, this fluctuating field will alter the center-of-mass diffusion of the chain, as described in section II.A, but the internal dynamics of the chain will remain unaffected. Notably, this is not the case if the chain monomers carry different charges. It can be shown (Appendix IV.D) that friction forces analogous to eq 7 arise under these conditions. The resulting GLEs for the chain are more complicated than eq 15 in that they exhibit hydrodynamic-like coupling between monomers, where the friction force on a given monomer depends on the velocity histories of other monomers. For a given nonuniform charge distribution along the chain, these equations in general require a numerical solution.

In general, the overall strength of electric friction from dipole tumbling remains moderate for intrinsically disordered proteins. Clearly, this might change if all sources of electric field fluctuations inside a cell are taken into account.

## III Conclusions

The variety of intermolecular interactions between macromolecules in the cellular interior is enormous, ranging from simple excluded volume to hydrophobic sticking to electrostatic effects. Here, we studied the effects of long-range electrostatic forces (as opposed to forces arising from direct physical contacts between macromolecules) on the diffusive dynamics of biopolymers. We described the complex cytosolic mixture of crowding proteins as a collection of diffusing and tumbling dipoles that cause a fluctuating electric field inside the cell. We obtained an analytical estimate of the magnitude of such field fluctuations and how these fluctuations impact the diffusion of a charged particle and the dynamics of a charged Rouse chain. To keep the problem analytically tractable, we treated electric field fluctuations as colored noise with a single characteristic time scale and an exponential memory kernel. The time scale is determined by the tumbling time of the dipoles. We show that the resulting friction coefficient is independent of the system size and proportional to the concentration of dipoles (i.e., the protein concentration in the cytosol). The friction coefficient also increases with the squared net charge of a diffusing particle, which causes a prominent slowdown in diffusion for highly charged particles. Similar effects were also found when investigating the behavior of a chain of charged beads in such a field. We find a significant effect of electric friction only if the net charge of the chain monomers exceeds an absolute charge of 10e.

Our numerical results provide only a crude estimate of electric friction effects. In reality, electrostatic fluctuations occur at multiple time scales and are caused not only by tumbling protein dipoles but also by other charged macromolecules such as RNA, charge regulation processes such as protonation and deprotonation of ionizable groups, and the translational diffusion of all charged species in the cytosol. However, our main goal was to provide an analytical framework to estimate electric friction. Our approach of describing the effect of electric field fluctuations on the diffusion of charged particles using GLEs is sufficiently flexible to account for additional noise sources. However, precise values for the strength of these fluctuations in realistic compositions of the cytosol are better obtained from molecular simulations.^11^ Our results suggest that even with the conservative estimate made in this work, electric friction in cells is on the verge of impacting the diffusion of highly charged particles and polymers without direct physical contacts.

## Appendix

### IV Appendix

#### IV.A Diffusion in the Presence of Electric Friction

To obtain the Stokes–Einstein relationship for the diffusion of a particle in the presence of white and colored noise, we write eq 4 in terms of velocities

A1

Using integration by parts, we obtain

A2

To remove the integral, we multiply eq A2 by *τ*_e_ ∂/∂*t* and add the result to eq A2, which reduces the integrodifferential equation to a second-order differential equation

A3

Using the Fourier transform (*t* → ω) defined by , we obtain the solution

A4

with the two Eigenvalues ω_1_ = −i/*τ*_e_ and ω_2_ = −i(*ξ*_s_ + *ξ*_e_)/*m* and the diffusion time scale *τ*_D_ = *m*/(*ξ*_s_ + *ξ*_e_). The Green–Kubo relationship (eq 3) is then given by

A5

The fluctuation–dissipation theorems after Fourier transform are given by and where the asterisk indicates the complex conjugate. With eq A4 and the identity *f*(ω) ≡ *f**(−ω), we then obtain for the velocity autocorrelation function

A6

Solving the integrals gives

A7

which results in the modified Stokes–Einstein relationship by inserting this expression into eq 3.

#### IV.B Electrostatic Dipole Potential with Salt Screening

Starting with eq 11, we can write the electric field for a single dipole

B1

where **n** = ∇*r* = **r**/*r* is the vector of unit length connecting the center of the dipole with the diffusing particle. From eq B1, it is easy to see that by averaging over the dipole orientation we obtain ⟨**d**⟩ = **0** and therefore ⟨**E**⟩ = **0**. To compute the mean-squared fluctuation, we compute **E**^2^

B2

Finally, with **d**^2^ = *d*^2^ and the angular average ⟨**dn**⟩ = *d*^2^/3, we obtain the expression in eq 12 by multiplying by the number of independent dipoles *N*. When performing the average in eq 12, we obtain for *R*^3^ ≫ *a*^3^

B3

Similarly, we obtain for the mean-square fluctuations of the electrostatic energy with *R* ≫ *a*

B4

#### IV.C Rouse Chain in the Presence of Electric Friction

We start with the fluctuation dissipation theorems for the two different noise processes.

C1

C2

Here, eq C1 accounts for the random force exerted by the solvent and eq C2 accounts for the fluctuations of the electrostatic force, which are assumed to have a characteristic time scale of *τ*_e_. Symbols δ and Δ are the Dirac and Kronecker delta functions, respectively. Note that the solvent and electrostatic random forces, *f*_s_(*k*, *t*) and *f*_e_(*k*′, *t*′), are assumed to be statistically independent

It is convenient to adopt periodic boundary conditions (i.e., *x*(*n* + *k*, *t*) ≡ *x*(*k*, *t*)).^16a,44^ After multiplying both sides of eqs C1 and C2 by e^i*qk*^ and summing over *k*, we obtain

C3

C4

which can also be written as

C5

C6

By multiplying both sides of eqs C5 and C6 by e^–i*ω*′*t*′^e^*iω*(*t*+t′)^ and integrating over *t* and *t*′, we obtain the time Fourier transforms

C7

C8

After reducing eq 19 to a second-order differential equation (Appendix A), we obtain

C9

Using time Fourier transform, we obtain eq 20 in the main text, which can be rewritten in terms of eigenvalues ω_1_ and ω_2_ as

C10

with  and . The end-to-end vector correlation function is defined by

C11

and using the definitions of Fourier transform we rewrite eq C11 as

C12

With the two noise processes (eqs C7 and C8) and the solutions (eq C10), we finally obtain

C13

Integration over ω′ and the summation of *q*′ gives, together with the definitions of *G*(*q*, ω) and *G*(−*q*, – ω),

C14

By solving the integrals and substituting *τ*_*j*_ = −i/*ω*_*j*_ with *j* = {1, 2}, we obtain

C15

with β = 1 + *ξ*_e_/*ξ*_s_.

#### IV.D Charged Rouse Chain in the Limit of Large Electric Field Correlation Lengths

We start by noting that the GLE (eq 4) can be derived from a “microscopic” model, in which coordinate *x* is coupled, via a Hookean spring, to a single fictitious overdamped “bath” degree of freedom *y*.^45^ The combined 2D system (*x*, *y*) undergoes Langevin dynamics without memory, but once *y* has been integrated out of the problem, the resulting dynamics of *x* alone obeys the GLE. Similarly, eq 15 can be derived by introducing a set of bath degrees of freedom *y*(*k*), with each harmonically coupled to the corresponding monomer coordinate *x*(*k*). Surrogate coordinates *y*(*k*) represent the degrees of freedom of the surroundings that couple to the chain via electrostatic forces.

When each monomer feels the same electric field, then their coordinates must couple to a single surrogate degree of freedom *y*, and the potential energy of the system is given by

D1

where *U*_R_ is the usual quadratic potential of the Rouse chain, γ_0_ is a coupling spring constant, and *c*_*k*_ is a dimensionless coefficient that quantifies the relative coupling of each bead to the electric field, which depends on the bead’s charge. Parameters γ_0_ and *c*_*k*_ will be related to the magnitude of the electric field fluctuations and to the monomer charges below.

The equations of motion for the combined system are the coupled Langevin equations

D2

and

D3

where *ξ*_*y*_ is a friction coefficient and *f*_*y*_ is the associated random noise for auxiliary mode *y*. Similar to the procedure described in ref (45), variable *y* can be integrated out of the problem, resulting in a system of coupled GLEs for the chain monomers

D4

where ψ(*k*, *t*) is a random force with a zero mean that satisfies the fluctuation–dissipation relationship

D5

and

D6

is the matrix of the friction kernels, with the memory time *τ*_e_ that can be expressed in terms of the parameters of the problem:

D7

Notice that if Γ_*kl*_ (*t*) → 0 for *k* ≠ *l*, then the Rouse model (eq 15) is recovered. Since the quantity *k*_B_*T*Γ_*kk*_ (0) is the mean squared amplitude of the electrostatic force acting on the *k*th charge (whose value we call *Q*_*k*_), we can write , and thus we can rewrite eq D6 as

D8

a result similar to eq 7, which expresses the memory kernels entering eq 4 in terms of the physical parameters of the chain and its surroundings.

For any specified set of charges *Q*_*k*_, finding the relaxation modes of the coupled equations of motion (eq D4 or, equivalently, finding the normal Langevin modes of eqs D2 and D3) can be accomplished numerically by reducing this to a linear algebra problem.^46^

If all monomer charges are identical, *Q*_*k*_ = *Q*, then the memory kernels  are identical for any *k* and *l*, and thus the friction forces felt by each monomer are also identical. The equations of motion for any relative distance *x*_*k*_ – *x*_*l*_ thus do not contain an electric friction, and thus this friction does not have any effect on the polymer’s internal dynamics. This conclusion is of course obvious: since all monomers feel exactly the same external force at any point of time, this force, while altering the motion of the chain’s centroid, has no effect on its internal dynamics.
